# Dietary Fiber Intake, Food Sources, and Their Associations with Metabolic Parameters in Adults

**DOI:** 10.3390/nu18162698

**Published:** 2026-08-18

**Authors:** Kamile Marakoğlu, Fatma Hümeyra Yerlikaya Aydemir, Zehra Doğruer, Esma Nakipoğlu, Abdullah Sivrikaya, Hadi Ziya Kavaklılar, Ülkü Sinem Koçak, Ayşegül Özkan, Beyza Nur Korkmaz, Mustafa Topkafa, Nazan Aktaş

**Affiliations:** 1 Department of Family Medicine, Faculty of Medicine, Selçuk University, 42075 Konya, Türkiye; esma.nakipoglu@selcuk.edu.tr (E.N.); zkavaklilar@gmail.com (H.Z.K.); sinemdanis8@gmail.com (Ü.S.K.); 2 Department of Medical Biochemistry, Faculty of Medicine, Selçuk University, 42075 Konya, Türkiye; fhumeyray@selcuk.edu.tr (F.H.Y.A.); zehraa4251@gmail.com (Z.D.); biyokaya@selcuk.edu.tr (A.S.); beyzanur_ozkann@hotmail.com (B.N.K.); 3 Department of Nutrition and Dietetics, Institute of Health Sciences, Selçuk University, 42075 Konya, Türkiye; dyt.aysegul.ozkan@gmail.com (A.Ö.); naktas@selcuk.edu.tr (N.A.); 4 Department of Chemistry and Chemical Processing Technologies, Vocational School of Technical Sciences, Konya Technical University, 42250 Konya, Türkiye; mtopkafa@ktun.edu.tr

**Keywords:** dietary fiber, insulin resistance, adult, cross-sectional studies

## Abstract

**Background/Objectives**: Dietary fiber intake is associated with lower risk of several chronic diseases; however, intake levels remain below recommended values in many populations. This study aimed to evaluate dietary fiber intake, major fiber sources, and their associations with sociodemographic and metabolic parameters in adults. **Methods**: This cross-sectional study included 820 adults (mean age 33.63 ± 13.58 years) attending the family medicine outpatient clinic. Dietary intake was assessed using a food frequency questionnaire. Fiber intake was evaluated as total intake and by source (whole-food and refined carbohydrate-rich foods). Sociodemographic, anthropometric, and metabolic data were also collected and analyzed. **Results**: Mean dietary fiber intake was 13.42 g/day, well below recommended levels. Fiber intake and its sources differed significantly across age groups (*p* < 0.05); younger adults consumed more fiber from refined carbohydrate-rich foods, whereas fruit- and vegetable-derived fiber increased with age. Women consumed more fiber from whole-food sources, whereas men consumed more fiber from refined carbohydrate-rich foods. Whole-food fiber intake was inversely associated with fasting insulin (r = −0.099) and Homeostatic Model Assessment of Insulin Resistance (HOMA-IR) (r = −0.094) in unadjusted analyses, but these associations were not significant after multiple-testing correction. In the adjusted model, total dietary fiber intake remained inversely associated with HOMA-IR (β = −0.148, *p* = 0.007). **Conclusions**: The findings highlight the importance of considering dietary fiber sources when evaluating associations between dietary fiber intake and metabolic parameters. Although associations between whole-food fiber intake and glycemic markers were attenuated after multiple-testing correction, total dietary fiber intake remained independently associated with HOMA-IR. Prospective longitudinal studies are needed to confirm these findings and clarify possible causal relationships.

## 1. Introduction

Dietary fiber refers to complex carbohydrates that cannot be broken down by human digestive enzymes in the small intestine and reach the colon, where they exert various physiological effects. It is found in whole grains, legumes, fruits, and vegetables [1]. Dietary fibers consist of monomers linked together by different glycosidic bonds. Therefore, they are organic polymers that exhibit heterogeneous and complex properties [2]. Dietary fibers are commonly classified as soluble and insoluble fibers according to their physicochemical properties. Soluble fibers dissolve in water and may be fermented by the gut microbiota, whereas insoluble fibers contribute primarily to fecal bulk and intestinal transit [3].

Adequate dietary fiber intake has been associated with a reduced risk of cardiovascular disease, type 2 diabetes, colorectal cancer, and weight gain [4]. Studies on this topic have been conducted in the literature. A systematic review published by Reynolds et al. in 2019 demonstrated that the risks of coronary heart disease, type 2 diabetes, and colorectal cancer, as well as mortality, were lower in individuals who consumed more dietary fiber [5]. Similarly, a prospective study published by Xu and colleagues in 2022 reported that all-cause mortality, as well as cardiovascular and cancer mortality, was lower in those who consumed more dietary fiber [6]. A review published in 2024 further demonstrated that dietary fiber has potential protective effects not only against chronic diseases but also against inflammation. It has been shown to have effects such as increased satiety, improved glycemic control, reduced cholesterol levels, and regulation of the microbiome [7].

The European Food Safety Authority (EFSA) recommends a daily dietary fiber intake of approximately 25 g for adults [8]. However, studies conducted in Europe and Turkey indicate that dietary fiber intake remains below recommended levels in a large proportion of the population [9,10]. Furthermore, considerable variation in fiber intake has been reported across different European countries and population groups [10].

According to data obtained from the Turkey Nutrition and Health Survey (TBSA/TNHS-2017), the average daily dietary fiber intake was found to be 24.4 g for men and 20.1 g for women. It was reported that fresh vegetable and fruit consumption was 415 g/day, legume consumption was 16.8 g/day, and oilseed/nut consumption was 9.9 g/day [11]. Turkish Dietary Guidelines (TÜBER) emphasizes that these food groups are primary sources of dietary fiber and that fiber intake across the general population remains below recommended levels. These studies indicate that consumption of fiber-rich food groups has not reached recommended levels across Turkey [9].

In Turkey, grains, and particularly bread, play a significant role in dietary fiber intake. According to large-scale studies conducted among adults, the most commonly consumed type of bread is white bread (87.3%), while consumption of whole-grain and whole-wheat bread is lower. Daily bread consumption was found to average 182.3 g for men and 124.5 g for women. Bread accounts for 27.1% of daily energy intake in men and 22.1% in women. However, total fiber intake often falls short of recommended levels [12].

Studies examining the level of knowledge and consumption habits regarding fiber-rich foods in Turkey indicate that individuals are aware of the positive health effects of fiber. According to a study by Yalçın and colleagues, fiber intake is influenced by factors such as age, sex, educational level, and living environment, and consumption of fiber-rich foods often falls short of recommended levels [1].

Although total dietary fiber intake has been widely studied in relation to metabolic health, findings on fiber obtained from different food sources remain limited. Previous studies have reported that associations with metabolic outcomes may differ according to the food source of dietary fiber, including fruits, vegetables, cereals, and other foods. These observations suggest that evaluating dietary fiber by food source may provide information that is not captured by total fiber intake alone [13,14].

A nationwide study conducted in Turkey showed that dietary habits vary according to sociodemographic characteristics such as age, sex, educational level, and place of residence [15]. Previous studies conducted in Konya have evaluated dietary habits among adolescents and university students and have reported suboptimal dietary patterns [16,17]. However, data on dietary fiber intake, dietary fiber sources, and their associations with metabolic parameters among adults in Konya remain limited. Therefore, evaluating dietary fiber intake and its food sources among adults in Konya, a province located in central Turkey with sociocultural characteristics and dietary habits that may broadly reflect those of the country, may complement national findings by providing region-specific evidence.

## 2. Materials and Methods

### 2.1. Study Design and Participants

This study was conducted at the Family Medicine Outpatient Clinic of Selçuk University Faculty of Medicine.

During the study period (from 1 March 2024 to 30 January 2025), 1104 adults aged 18–65 years who attended the Family Medicine outpatient clinic and agreed to complete the sociodemographic data form and the Food Frequency Questionnaire (FFQ) were recruited. Of these, 284 were excluded: 34 were older than 65 years, 60 were using three or more medications, 10 were pregnant, 55 had chronic diseases meeting the exclusion criteria, 50 had missing height or weight data, and 75 had incomplete questionnaire data. Consequently, 820 participants were included in the final analysis (Figure S1). The final study population comprised 820 adults (569 women and 251 men), with a mean age of 33.63 ± 13.58 years (range: 18–65 years).

### 2.2. Ethics Approval and Consent to Participate

The study was approved by the Local Ethics Committee of Selçuk University Faculty of Medicine (Decision No. 2024/254-2026/137) and was conducted in accordance with the Declaration of Helsinki. Written informed consent was obtained from all participants before enrollment in the study.

### 2.3. Data Collection

Participants’ sociodemographic characteristics, lifestyle factors, anthropometric measurements, metabolic parameters, and dietary habits were obtained using structured questionnaire forms and clinical assessment records.

Height was measured using a seca 264 digital stadiometer (seca GmbH & Co. KG, Hamburg, Germany) with the participants barefoot. Body weight, body fat mass, fat-free mass, body mass index (BMI), visceral fat area (VFA), and waist-to-hip ratio were assessed using the InBody 770 bioelectrical impedance analyzer (Biospace Co., Seoul, Republic of Korea).

The InBody 770 device measured the visceral fat area representing the visceral fat tissue content as normal below 100 cm^2^, with more than 100 cm^2^ as high. The device is 98.4% compatible with DEXA in body composition measurement (InBody 770 instruction manual). Before each measurement, participants’ age and sex were entered into the device. Body composition parameters, including total body water, protein mass, mineral content, fat mass, and lean body mass, were measured using the multi-frequency InBody 770 analyzer (1, 5, 50, 250, 500, and 1000 kHz) according to the manufacturer’s instructions. Measurements were performed with participants standing barefoot after removing shoes and heavy outer clothing [18]. All assessments were conducted in the morning following an overnight fast of at least 8 h. After the test, the data was recorded on the device and printed.

Venous blood samples were collected in the morning after an overnight fast as part of the routine clinical evaluation. Fasting plasma glucose, triglyceride, and total cholesterol concentrations were measured using spectrophotometric methods on a Beckman Coulter AU5800 automated chemistry analyzer (Beckman Coulter, Inc., Brea, CA, USA), whereas fasting serum insulin concentrations were measured by electrochemiluminescence immunoassay (ECLIA) using a Roche Cobas e601 analyzer (Roche Diagnostics GmbH, Mannheim, Germany) at the Central Laboratory of Selçuk University Faculty of Medicine. Glucose, triglyceride, and total cholesterol concentrations were expressed in mg/dL, while insulin concentrations were expressed in µIU/mL. HOMA-IR values were automatically generated in the hospital laboratory information system using fasting plasma glucose and fasting serum insulin concentrations according to the following formula: HOMA-IR = fasting plasma glucose (mg/dL) × fasting serum insulin (µIU/mL)/405. The denominator of 405 corresponds to 22.5 multiplied by the glucose conversion factor of 18, accounting for the expression of glucose in mg/dL rather than mmol/L [19]. No additional unit conversion was required for insulin concentrations.

### 2.4. Evaluation of Food Consumption Data and Food Classification

Participant dietary intake was evaluated using a Food Frequency Questionnaire (FFQ) specifically adapted to reflect Turkish dietary patterns and traditional culinary culture. The FFQ was administered via face-to-face interviews by trained dietitians to standardize data collection and reduce recall bias. The questionnaire assessed dietary habits over a reference period of the past 1 month and comprised 75 food items. For each food item, participants reported their consumption frequency across 7 categories (ranging from “Never” to “Every day”) along with standard portion sizes (e.g., cups, slices, grams, pieces). Although a separate pilot validity and reliability study was not conducted specifically for this sample, the questionnaire items were adapted from published population-based dietary studies in Turkey to ensure cultural appropriateness [20,21].

In this study, while assessing participants’ dietary habits and daily fiber intake, the foods consumed were divided into two main categories “whole food sources” and “refined carbohydrate-rich foods” based on the degree of industrial processing they underwent. This classification was based on TÜBER and the internationally recognized NOVA food classification system [9,22].

Accordingly, the grouping used in this study is as follows:

Whole Food Sources (Unprocessed/Minimally Processed Foods): These include foods whose natural cellular structure, germ, bran, and fiber matrices remain intact. This group includes whole grains and unrefined grain products such as whole-wheat bread, oats, flaxseed, bulgur, and whole-wheat pasta; oilseeds such as walnuts, almonds, and hazelnuts; legumes such as dried beans, chickpeas, and lentils; and all types of fresh and dried vegetables and fruits.

Refined Carbohydrate-Rich Foods (Processed/Ultra-Processed Foods): These include products subjected to mechanical or industrial refining where bran and germ are removed, or commercial mixed items. This category includes fast-food items (e.g., pizza, hamburgers, lahmacun), white bread, pastries, and cookies, as well as **refined grain products such as white pasta and white rice** (where industrial milling removes the bran and germ, significantly lowering dietary fiber content).

Food Items and Categorization for Fiber Analysis:

To evaluate dietary fiber consumption patterns, a total of 44 fiber-containing food items selected from the comprehensive 75-item FFQ were analyzed. These items were grouped into 5 main food categories based on their nutritional characteristics, as detailed below:

- Bread and grains: Whole-wheat bread, oats, flaxseed, whole-wheat pasta, bulgur.
- Nuts: Walnuts, almonds, hazelnuts, etc.
- Dried legumes: Dried beans, chickpeas, kidney beans, lentils and other dried legumes.
- Fruits and vegetables: potatoes, Jerusalem artichokes, leeks, onions, garlic, broad beans, black-eyed peas, asparagus, artichokes, tomatoes, green leafy vegetables, fresh beans, peas, red beets, mushrooms, bananas, blueberries, pineapples, red plums, pomegranates, grapes, mangoes, dried fruits and other fruits.
- Processed: Pizza, hamburger, lahmacun, white bread, pastries, rice, cookies.

Portion Conversions and Calculation of Daily Intake:

The Nutrition Information System (eBeBiS, version 9.0; https://www.ebebis.com.tr, accessed on 12 August 2026) software was used to calculate daily energy, macronutrient (carbohydrate, protein, fat), and dietary fiber intakes by converting food consumption reports obtained from participants into standard gram measurements. During the calculation process, participants’ non-standard quantity reports (bowl, slice, spoon, piece, etc.) were first converted to standard portion sizes (g/mL) using the Food and Nutrition Photo Catalog as a reference [23]. Subsequently, these obtained gram amounts were multiplied by appropriate conversion factors based on the participant’s frequency of consumption of the relevant food to convert them into “daily average gram amounts.” The specific macronutrient and fiber contents of these foods, for which daily consumption amounts were determined, were obtained directly from the eBeBİS database. The net fiber contribution and macronutrient values of each specific food consumed by the participants (e.g., legumes, whole grains, or vegetables) were analyzed via the program and incorporated into the participants’ total daily intake profiles, thereby preparing the data for statistical evaluation. Minor differences between total dietary fiber values and the sum of soluble and insoluble fiber fractions may occur because these values are derived from different nutrient composition records within the database. These differences reflect database characteristics rather than errors in data processing.

### 2.5. Statistical Analysis

The statistical analysis of the research data was conducted using IBM SPSS Statistics for Windows, version 22.0 (IBM Corp., Armonk, NY, USA). The choice of statistical tests depended on the distribution of the analyzed variables. Normality of distribution was assessed using the Shapiro–Wilk test. Descriptive statistics were presented using mean ± standard deviation, median [1st and 3rd quartiles], or frequency (*n*) and percentage (%) values, depending on the distribution characteristics of the data. After assessing the normality of the data distribution, parametric or non-parametric tests appropriate for the data structure were used to compare independent groups. The direction and strength of the relationships between variables were evaluated using Spearman’s correlation analysis. To account for multiple comparisons, *p*-values obtained from correlation analyses were adjusted using the Benjamini–Hochberg false discovery rate (FDR) procedure. In addition, multiple linear regression analysis was performed to evaluate the independent association between dietary fiber intake and HOMA-IR after adjustment for age, sex, BMI, and total energy intake. A significance level of *p* < 0.05 was adopted for all statistical analyses.

## 3. Results

No statistically significant difference was found between the mean ages and body mass indices (BMI) of female and male participants (*p* > 0.05). However, women’s visceral fat area (115.83 ± 48.17 cm^2^) was found to be significantly higher than that of men (89.85 ± 41.57 cm^2^) (*p* < 0.001). No significant sex differences were observed in glucose, total cholesterol, insulin, or HOMA-IR levels. However, triglyceride levels were significantly higher in men than in women (133.28 ± 85.62 vs. 99.99 ± 82.72 mg/dL, *p* < 0.001). It was observed that 69.8% of the participants did not smoke, 90.7% did not consume alcohol, 85.7% lived in a nuclear family, and 33.8% held a bachelor’s degree (Table 1).

Average daily total dietary fiber intake was 13.42 g. Average daily energy, carbohydrate, protein, and fat intakes were 1653.04 kcal, 162.22 g, 56.80 g, and 85.42 g, respectively. When the components of dietary fiber were examined, the average soluble fiber intake was 5.14 g, and the insoluble fiber intake was 8.51 g. The average nutrient intake from whole food sources was 9.39 g for fiber and 1071.6 kcal for energy (Table 2).

Statistically significant differences were observed in the intake of total fiber, calories, protein, fat, and insoluble and soluble fiber from whole food sources across age groups (*p* < 0.05). When the nutrient values obtained from refined carbohydrate-rich foods were examined by age group, no statistically significant differences were found in total fiber, insoluble fiber, and soluble fiber intake (*p* > 0.05). However, age-related significant differences were detected in total calorie (*p* < 0.001), carbohydrate (*p* < 0.001), protein (*p* = 0.005), and fat (*p* < 0.001) intakes. When total nutrient intakes were analyzed by age group, statistically significant differences were found in total fiber (*p* = 0.005), carbohydrate (*p* = 0.002), fat (*p* = 0.001), and insoluble fiber (*p* = 0.002) intakes. In contrast, no age-related differences were observed in total calorie (*p* = 0.259), protein (*p* = 0.317), and soluble fiber (*p* = 0.135) intake (Table 3).

Although women showed numerically higher intakes of total, soluble, and insoluble dietary fiber, these differences were not statistically significant (*p* > 0.05). However, significant differences in total energy, protein, and fat intake were observed between the sexes. Although fiber intake from refined carbohydrate-rich foods was found to be higher in men, no significant difference was observed in fiber intake (*p* > 0.05). Significant differences were found between sexes in total calorie (*p* = 0.003), protein (*p* = 0.013), and fat (*p* < 0.001) intake (Table 4).

**Figure 1:**
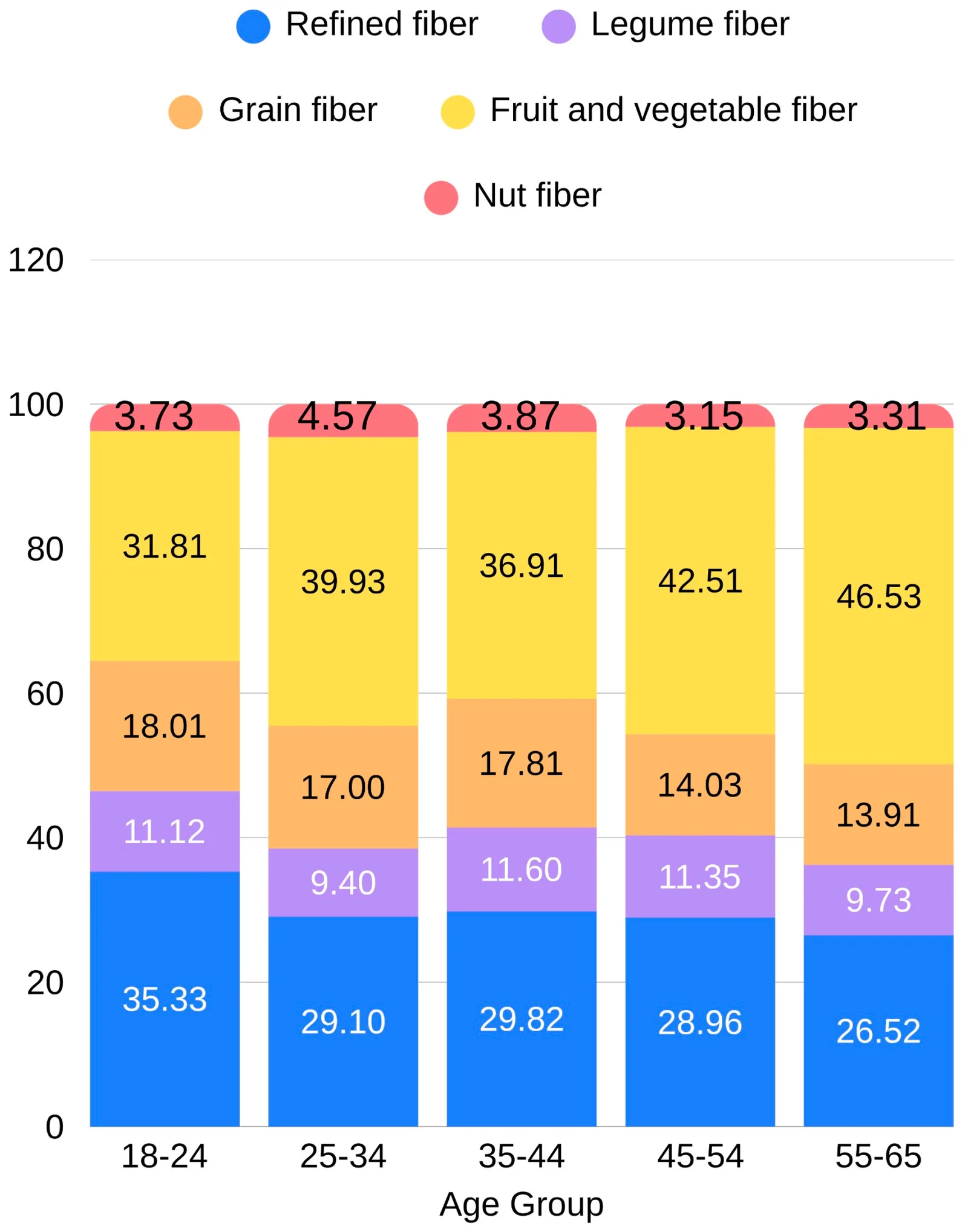
Proportional distribution of dietary fiber intake sources by age groups.

The proportional distribution of dietary fiber sources differed across age groups (Figure 1).

Looking at the overall distribution, the most striking finding is the opposite trend in the consumption of fiber from fruits and vegetables versus fiber derived from refined carbohydrate-rich foods. In the youngest group (ages 18–24), fiber derived from refined carbohydrate-rich foods accounted for the largest portion of dietary fiber at 35.33%, while this percentage was observed to decrease gradually with age, dropping to 26.52% in the 55–65 age group. In contrast, while fruit and vegetable fiber intake was 31.81% in the 18–24 age group, it increased steadily with age, becoming the dominant fiber source at 46.53% in the 55–65 age group.

When other dietary fiber sources were examined, the following results were obtained:

- Bread and Grain Fiber: A general downward trend in consumption was observed with increasing age (from 18.01% in the 18–24 age group to 13.91% in the 55–65 age group).
- Legume Fiber: Consumption remained relatively stable across all age groups, with the lowest rate observed in the 25–34 age group (9.40%) and the highest in the 35–44 age group (11.60%).
- Nuts: This was the source contributing the least to total daily fiber intake across all age categories, with rates varying within a narrow range of 3.15% to 4.57%.

Differences across age groups were evaluated using one-way ANOVA. Fruit and vegetable fiber intake differed significantly across age categories (p<0.001), whereas differences for legume fiber (p=0.067), grain fiber (p=0.418), nut fiber (p=0.431), and refined fiber (p=0.389) were not statistically significant.

**Figure 2:**
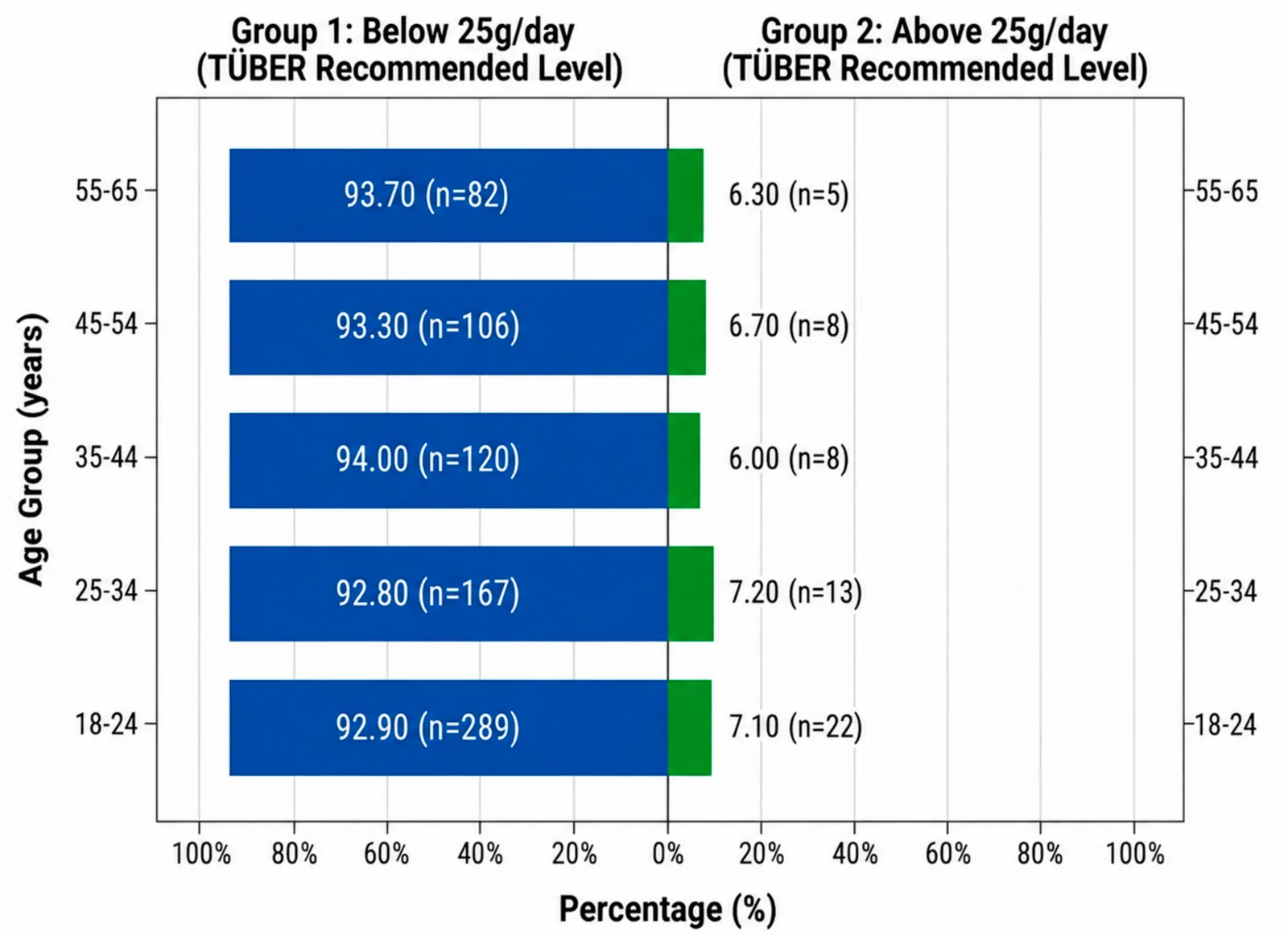
Proportion of individuals meeting the recommended daily dietary fiber intake (≥25 g) across age groups. (1) Intake below 25 g/day; (2) intake ≥ 25 g/day according to TÜBER recommendations.

Although total dietary fiber intake (in grams) showed a statistically significant progressive increase with advancing age (Table 3; p=0.005), this quantitative increase was insufficient to bridge the gap toward national nutritional recommendations. When daily fiber intakes were evaluated categorically against the 25 g/day target recommended by TÜBER, the vast majority of participants across all age brackets (92.8–94.0%) remained below this reference threshold. The proportion of individuals achieving the recommended intake of at least 25 g/day ranged between only 6.0% and 7.2% across age categories (Figure 2). A Pearson Chi-square test revealed no statistically significant difference across age groups ($\chi^{2}=0.233$, df=4, p=0.994). Taken together with the median intake values in Table 3, these findings demonstrate that despite higher absolute intake among older adults, dietary fiber deficit represents a widespread, population-level public health issue spanning all adult age groups.

The relationships between participants’ dietary fiber intake sources and glycemic control parameters (fasting glucose, insulin, HOMA-IR, and glycated hemoglobin [HbA1c]) were evaluated using Spearman’s correlation analysis (Table 5).

While unadjusted bivariate correlation analyses initially suggested inverse associations between whole-food fiber intake and both HOMA-IR (r=−0.094,p=0.024) and fasting insulin (r=−0.099,p=0.015), these correlations did not retain statistical significance after adjusting for multiple testing via the Benjamini–Hochberg False Discovery Rate (FDR) procedure (q=0.096 for both).

The observed associations differed according to the dietary source of fiber. According to the statistical analysis, a statistically significant negative correlation was found only between dietary fiber intake derived from whole food sources and fasting insulin levels and the HOMA-IR index, a marker of insulin resistance. In contrast, no significant correlation was found between total fiber intake and fasting glucose, insulin, HOMA-IR, or HbA1c. Following adjustment for multiple testing using the Benjamini–Hochberg false discovery rate procedure, the associations between whole-food fiber intake and both insulin and HOMA-IR were no longer statistically significant (adjusted *p* = 0.096), suggesting that the observed correlations may be sensitive to multiple-testing correction. However, because insulin resistance may be influenced by several demographic and metabolic factors, a multiple linear regression analysis was performed to further examine the relationship between total dietary fiber intake and HOMA-IR. Total dietary fiber intake remained inversely associated with HOMA-IR after adjustment for age, sex, BMI, and total energy intake (B=−0.014, SE=0.005, β=−0.148, p=0.007). The final model explained 15.8% of the variance in HOMA-IR ($R^{2}=0.158$, Adjusted $R^{2}=0.151$) (Table 6).

Additionally, no significant correlation was observed between fiber derived from whole food sources or total fiber intake and visceral fat area or BMI (*p* > 0.05) (Table 7).

## 4. Discussion

The EFSA recommends a daily dietary fiber intake of approximately 25 g for adults. However, in Europe and Turkey, intake in most individuals does not reach this level [8,9,10,24]. A study conducted in Greece reported that daily dietary fiber intake in adults was approximately 18 g/day for men and 14 g/day for women, both of which fell below the recommended levels [25]. Similarly, a study conducted in Australia showed that the median fiber intake among adults was 20.7 g/day, while in the United States, the average fiber intake was approximately 17 g/day, and fiber intake in both populations was found to be below the recommended levels [26,27]. In our study, the average daily total dietary fiber intake was found to be 13.42 g; this value is below the recommended daily fiber intake [8,9,10]. This level of fiber intake is an indicator of the dietary characteristics of the geographic region located in central Turkey and its local population. Furthermore, it was found that the vast majority of individuals across all age groups (92.8–94.0%) fell short of the daily 25-g fiber target, with only 6.0–7.2% meeting the recommended level. These findings indicate that inadequate dietary fiber intake was common among adults included in this study. Compared with national survey data, the lower dietary fiber intake observed in our study may reflect differences in the characteristics of the study population.

Previous studies have identified poor diet as a major modifiable risk factor for noncommunicable diseases [5,28]. In a 2019 study by Reynolds and colleagues, it was reported that as dietary fiber intake increased, the risk of coronary heart disease, type 2 diabetes, and colorectal cancer decreased. High fiber intake has been associated with significant reductions in body weight, systolic blood pressure, and total cholesterol levels, with the greatest benefits observed at approximately 25–29 g/day of fiber intake [5]. These beneficial effects may be explained by several mechanisms, including delayed gastric emptying, stimulation of satiety hormones, modulation of glucose and lipid metabolism, and regulation of metabolic responses through the gut microbiota [29]. In addition, higher dietary fiber intake has been associated with lower risks of abdominal obesity, metabolic syndrome, and hypertension [13,30]. However, the low average dietary fiber intake observed in our study indicates that most participants did not reach the dietary fiber intake level at which health benefits have been reported.

In a study conducted by Yalçın and colleagues in the Turkish population, it was shown that participants possessed a certain level of knowledge about dietary fiber; however, their regular consumption of fiber-rich foods was limited [1]. This highlights the discrepancy between knowledge levels and daily dietary habits. Similarly, the low daily dietary fiber intake observed in our study suggests that inadequate fiber intake was common among adults in this study population.

When participants were analyzed according to age group, a significant difference was observed between groups in terms of total fiber intake (*p* = 0.005). The lowest total fiber intake was observed in the 18–24 age group, while fiber intake increased in older age groups, particularly after age 35. Additionally, significant differences were found among age groups regarding total fiber, energy, protein, fat, soluble fiber, and insoluble fiber intakes derived from whole food sources (*p* < 0.05). In contrast, fiber intake from refined carbohydrate-rich foods did not show significant differences across age groups (*p* = 0.103). When the distribution of fiber sources was examined, fiber derived from refined carbohydrate-rich foods accounted for the largest portion of dietary fiber (35.33%) in the 18–24 age group. This proportion gradually declined with age, reaching 26.52% in the 55–65 age group, whereas the proportion of fiber derived from fruits and vegetables increased from 31.81% to 46.53%. These findings suggest that both the amount and the dietary sources of fiber differed across age groups. Similarly, a study conducted among adults in Greece reported that older individuals consumed more fruits, vegetables, legumes, and fish compared to younger individuals, while younger individuals consumed more grains, sugary products, and various beverages [25]. TÜBER data indicate that fiber intake is generally insufficient in the population and that the proportion of individuals reaching the recommended level is lower in older age groups [9]. Although total dietary fiber intake increased across age groups in our study, most participants in every age group still consumed less than the recommended 25 g/day. Therefore, the observed statistically significant differences between age groups may have limited clinical relevance. This interpretation is supported by the observation that only 6.0–7.2% of participants across all age groups met the recommended dietary fiber intake (≥25 g/day), indicating that inadequate fiber intake remained common regardless of age.

In analyses stratified by sex, no significant differences were observed between women and men in terms of total fiber, soluble fiber, and insoluble fiber intake from whole food sources (*p* > 0.05). In contrast, significant differences in energy, protein, and fat intake from whole food sources were observed between sexes. Similarly, significant differences were observed between sexes in energy, protein, and fat intake from refined carbohydrate-rich foods; however, no significant differences were observed in fiber intake from refined carbohydrate-rich foods. These findings indicate that no significant sex differences were observed in dietary fiber intake, whereas the observed differences were primarily related to total energy, protein, and fat intake.

In contrast, TÜBER 2022 data indicate that a higher proportion of men in the general population consume fiber at adequate levels. Indeed, the proportion of individuals aged 18 and older who consume fiber at or above the adequate intake level was reported as 40.8% for men and 23.9% for women [9]. This discrepancy may be reflect the fact that our study evaluated fiber intake based on daily amounts, whereas the TÜBER study focused on the proportion of individuals meeting the adequate intake level. Additionally, differences in sample composition and dietary habits may have contributed to this outcome.

Although an inverse correlation was observed between whole-food fiber intake and markers of insulin resistance, no significant association was found between total fiber intake and these parameters. However, the inverse correlations did not remain statistically significant after correction for multiple testing and should therefore be interpreted cautiously. In the adjusted regression model, total dietary fiber intake remained independently and inversely associated with HOMA-IR after controlling for age, sex, BMI, and total energy intake (β = −0.148, *p* = 0.007). Moreover, the regression model explained 15.8% of the variance in HOMA-IR. Nevertheless, our findings are broadly consistent with those reported by Tucker et al. A 2018 study by Tucker et al. involving 6374 adults reported that higher dietary fiber intake was associated with lower insulin resistance. When participants were classified according to the recommended dietary fiber intake, HOMA-IR values differed significantly between groups [31]. Similarly, a 2021 study conducted on Korean adults reported that cardiovascular risk factors may vary not only based on total dietary fiber intake but also depending on the dietary source of fiber. This study demonstrated that, in men, total fiber and fruit-derived fiber intake were inversely associated with the prevalence of metabolic syndrome. In women, a diet richer in fruit fiber was found to be associated with lower prevalence of obesity, abdominal obesity, hypertension, and type 2 diabetes. These findings suggest that the potential protective effects of dietary fiber may depend not only on total intake but also on its food source [13]. However, these findings should be compared with caution because of differences in study populations, dietary assessment methods, and study design.

Studies in the literature have shown that increased dietary fiber intake is associated with a reduced risk of obesity, insulin resistance, prediabetes, and type 2 diabetes [13,31,32,33]. In a study analyzing data from the National Health and Nutrition Examination Survey (NHANES) in the United States from 1999 to 2018, it was reported that the incidence of obesity was significantly lower in groups with high daily fiber intake. In this study, dietary data were collected using two separate 24-h dietary recall methods; BMI, waist circumference, and various demographic variables were evaluated together. It was found that the risk of obesity in the group with the highest fiber intake was 44% lower compared to the group with the lowest fiber intake. Additionally, it was determined that every 10-g increase in daily fiber intake was associated with a significant reduction in the likelihood of obesity. This association was observed similarly in men and women, across different ethnic groups, and in all age categories [32]. A 2018 study of African American women reported that each 1-g increase in dietary fiber intake was associated with a significant decrease in BMI during the follow-up period [34]. Additionally, studies conducted among Japanese adults have shown an inverse association between dietary fiber and visceral fat [35,36]. A study conducted in Australia also noted that as grain consumption increased, dietary fiber intake rose, and this was associated with lower BMI and waist circumference [37]. Ozato and colleagues demonstrated that an increase in soluble fiber intake is inversely associated with changes in visceral fat area [35]. In contrast, our study did not find a significant association between total fiber or whole food derived fiber and BMI or visceral fat area. This finding may reflect differences in sample characteristics, as well as variations in the amount and source of fiber. In addition, the mean BMI of both women and men exceeded 25 kg/m^2^, indicating that the study population was predominantly overweight. Therefore, the present findings should be interpreted within the context of this study population.

Similarly, a study conducted among overweight/obese Iranian adolescents reported that higher dietary fiber intake was associated with a reduced risk of hyperglycemia [38]. Furthermore, a study conducted among adults in China demonstrated that fiber intake in the general population was generally low and that no significant association was found between total fiber intake and many chronic diseases. In the same study, findings differed according to the dietary fiber source, suggesting that dietary fiber should be evaluated not only in terms of total intake but also according to its food sources [14]. In our study, no significant association was observed between total dietary fiber intake and most glycemic parameters. Although inverse correlations were observed between whole-food fiber intake and fasting insulin and HOMA-IR, these associations did not remain statistically significant after correction for multiple testing. Therefore, these associations should be interpreted with caution and require confirmation in future prospective studies.

Several methodological limitations should be considered when interpreting the findings of this study. First, dietary intake was assessed using a Food Frequency Questionnaire (FFQ), which relies on self-reported data and is therefore subject to recall bias and measurement errors. To minimize these limitations, participants were guided using visual portion-size catalogs, and a standardized approach based on the predominant ingredient was applied to classify complex food items. Second, the cross-sectional design of the study precludes the determination of causal relationships and temporal changes between dietary fiber sources and metabolic and body composition parameters. Residual confounding due to unmeasured lifestyle factors such as physical activity cannot be excluded. Finally, because the study was conducted in a single center and within a single geographic region, the generalizability of the findings to the broader population may be limited. These findings suggest that the quality and source of dietary fiber may be at least as important as the total amount consumed when evaluating metabolic health.

## 5. Conclusions

The findings indicate that daily dietary fiber intake among adults remains below recommended levels. Total fiber intake increases with age. However, this increase does not reach the recommended level. Furthermore, the source of fiber intake also changes with age. Younger individuals have higher dietary fiber intake from refined carbohydrate-rich foods, whereas older age groups have higher dietary fiber intake from fruits and vegetables. While no significant difference in fiber intake was observed between sexes, differences were found in energy, protein, and fat intake. Inverse correlations were observed between fiber intake from whole food sources and fasting insulin and HOMA-IR; however, these associations did not remain statistically significant after correction for multiple testing. The findings highlight the importance of considering dietary fiber sources when evaluating associations between dietary fiber intake and metabolic parameters. Future studies should evaluate dietary fiber according to its food sources in addition to total dietary fiber intake. Public health strategies should emphasize not only increasing total dietary fiber intake but also promoting fiber consumption from minimally processed whole-food sources such as fruits, vegetables, legumes, and whole grains. However, prospective studies are needed to make definitive recommendations.

## Figures and Tables

**Table 1:** Demographic and Metabolic Parameters of the Participants.

	Female Subjects (*n* = 569) Mean ± SD *n* (%)	Male Subjects (*n* = 251) Mean ± SD	*p*-Value
**Age (Years)**	33.99 ± 13.70	32.85 ± 12.54	0.267
**Body Mass Index (kg/m^2^)**	25.90 ± 5.58	26.22 ± 4.19	0.439
**Fat Percentage**	35.44 ± 8.23	26.13 ± 8.20	<0.001
**Fat Area (cm^2^)**	115.83 ± 48.17	89.85 ± 41.57	<0.001
**Metabolic Parameters**			
**Glucose (mg/dL)**	90.94 ± 17.89	91.07 ± 11.15	0.922
**Triglycerides (mg/dL)**	99.99 ± 82.72	133.28 ± 85.62	<0.001
**Cholesterol (mg/dL)**	181.20 ± 40.64	181.18 ± 39.84	0.995
**Insulin (µIU/mL)**	11.32 ± 8.04	12.40 ± 9.05	0.129
**HOMA-IR**	2.50 ± 1.87	2.71 ± 1.94	0.21
**Tobacco use**			
**Current smoker**		131 (16.0)	
**Occasional smoker**		62 (7.6)	
**Ex-smoker**		54 (6.6)	
**Non-smoker**		573 (69.8)	
**Alcohol use**			
**Yes**		76 (9.3)	
**No**		744 (90.7)	
**Marital Status**			
**Married**		387 (47.2)	
**Single**		433 (52.8)	
**Family Type**			
**Nuclear family**		703 (85.7)	
**Extended family**		76 (9.3)	
**I live alone**		41 (5)	
**Level of education**			
**Primary school**		98 (11.9)	
**Secondary school**		30 (3.7)	
**High school**		240 (29.2)	
**Associate degree**		58 (7.1)	
**Bachelor’s degree**		277 (33.8)	
**Master’s degree**		117 (14.3)	

**Table 2:** Comparison of Total Dietary Fiber and Nutrient Intake and Intake Derived from Whole Food Sources.

Variables (*n* = 820)	Total Intake Mean ± SD	Whole Food Intake Mean ± SD
**Dietary Fiber (g)**	13.42 ± 7.50	9.39 ± 5.88
**Energy (kcal)**	1653.04 ± 799.21	1071.6 ± 512.31
**Carbohydrate (g)**	162.22 ± 107.32	66.89 ± 37.32
**Protein (g)**	56.80 ± 28.27	42.40 ± 22.36
**Fat (g)**	85.42 ± 43.19	70.09 ± 39.95
**Soluble Fiber (g)**	5.14 ± 3.05	3.1 ± 2.04
**Insoluble Fiber (g)**	8.51 ± 4.67	6.24 ± 3.94

Values are presented as mean ± standard deviation (SD).

**Table 3:** Dietary Fiber and Nutrient Intake by Age Groups.

Variables	18–24 (*n* = 311) (IQR) Median	25–34 (*n* = 180) (IQR) Median	35–44 (*n* = 128) (IQR) Median	45–54 (*n* = 114) (IQR) Median	55–65 (*n* = 87) (IQR) Median	*p*-Value
**Dietary Fiber (g)**						
Whole foods	[4.8–10.8] ^a^ 6.8	[5.2–11.6] 7.5	[5.9–14.0] 9.0	[5.9–11.6] 8.5	[7.3–13.9] ^b^ 8.9	<0.001
Refined foods	[1.2–5.9] 2.8	[0.71–4.97] 1.96	[0.8–4.8] 2.4	[0.7–4.8] 2.9	[0.7–5.4] 2.5	0.103
Total	[7.4–16.2] 11.4	[7.1–16.2] 11.5	[9.5–18.4] 13.9	[8.9–16.2] 12.1	[9.5–18.4] 14.0	0.005
**Energy (kcal)**						
Whole foods	[637.6–1160.0] 866.4	[721.1–1369.3] 998.5	[832.9–1478.8] 1065.3	[721.5–1262.2] 1005.3	[886.7–1433.9] 1145.9	<0.001
Refined foods	[207.9–952.3] 473.7	[126.6–702.6] 298.3	[107.9–768.7] 320.2	[117.8–506.2] 285.3	[118.6–570.8] 284.7	<0.001
Total	[1105.5–2055.2] 1452.7	[1065.9–2124.7] 1507.4	[1244.3–2043.6] 1639.7	[1008.6–1838.3] 1428.4	[1197.3–1989.4] 1508.1	0.259
**Carbohydrate (g)**						
Whole foods	[40.9–82.2] 56.8	[43.5–85.1] 58.4	[44.9–85.3] 61.2	[40.1–73.7] 55.8	[43.7–86.0] 61.2	0.407
Refined foods	[34.6–156.5] 74.6	[18.3–111.3] 47.6	[17.9–123.2] 52.4	[20.0–89.1] 48.6	[16.2–100.8] 46.0	<0.001
Total	[101.4–232.3] 145.6	[88.3–202.0] 129.7	[89.8–208.6] 142.5	[79.8–152.0] 114.2	[81.4–178.0] 128.0	0.002
**Protein (g)**						
Whole foods	[25.5–49.9] 34.8	[28.5–56.1] 39.1	[30.5–53.0] 40.1	[28.0–48.0] 36.4	[32.9–50.6] 41.6	0.014
Refined foods	[4.6–21.5] 10.3	[2.6–16.2] 6.8	[2.3–18.2] 7.5	[2.9–13.8] 8.0	[2.3–16.5] 7.0	0.005
Total	[37.0–68.4] 51.0	[37.8–72.6] 53.2	[40.4–66.5] 54.3	[35.7–65.5] 46.5	[42.7–66.4] 53.0	0.317
**Fat (g)**						
Whole foods	[35.2–76.5] 50.9	[44.0–90.6] 60.2	[53.0–96.4] 73.0	[47.5–88.2] 68.3	[60.3–102.0] 75.5	<0.001
Refined foods	[5.1–24.4] 12.9	[3.2–17.8] 7.8	[2.9–18.0] 7.9	[2.2–11.9] 4.6	[2.2–12.5] 4.9	<0.001
Total	[51.3–97.7] 70.3	[53.1–113.0] 79.1	[65.5–113.0] 85.2	[53.7–98.9] 79.3	[66.3–114.5] 91.3	0.001
**Soluble Fiber (g)**						
Whole foods	[3.2–7.2] 4.5	[3.4–8.0] 5.0	[4.0–9.4] 5.8	[3.9–7.8] 5.6	[4.9–9.8] 6.0	<0.001
Refined foods	[0.6–2.9] 1.4	[0.4–2.4] 1.0	[0.4–2.4] 1.2	[0.3–2.2] 1.4	[0.4–2.8] 1.2	0.084
Total	[2.8–6.5] 4.5	[2.7–6.3] 4.4	[3.4–6.7] 5.1	[3.2–5.9] 4.3	[3.3–7.0] 5.2	0.135
**Insoluble Fiber (g)**						
Whole foods	[1.6–3.5] 2.3	[1.7–3.8] 2.4	[1.9–4.6] 3.2	[1.88–3.76] 2.87	[2.2–4.4] 2.9	<0.001
Refined foods	[0.6–2.9] 1.5	[0.3–2.6] 1.0	[0.4–2.5] 1.2	[0.3–2.5] 1.5	[0.3–2.8] 1.4	0.129
Total	[4.7–10.2] 6.9	[4.7–10.5] 7.2	[5.9–11.7] 8.9	[5.5–10.5] 7.6	[6.3–11.4] 8.6	0.002

Values are presented as Median (IQR). *p*-values were calculated using the Kruskal–Wallis test. Post-hoc pairwise comparisons using the Dunn–Bonferroni test indicated that age-related differences across total, whole-food, and refined nutrient/fiber intake parameters were consistently driven by significantly distinct consumption patterns in the youngest age group (18–24 years) compared to older age categories (particularly 35–44, 45–54, and 55–65 years; p<0.05). Values with different superscript letters (a, b) within the same row differ significantly according to the Dunn–Bonferroni post hoc test (*p* < 0.05).

**Table 4:** Sex-Based Comparison of Dietary Fiber Intake and Nutrients.

	Female Subjects (*n* = 569) Median (IQR)/Mean ± SD	Male Subjects (*n* = 251) Median (IQR)/Mean ± SD	*p*-Value
**Dietary Fiber (g)**			
Whole foods	8 [5.5–11.7]	7.8 [4.7–12.2]	0.228
Refined foods	3.7 ± 3.7	5.6 ± 5.7	<0.001
**Energy (kcal)**			
Whole foods	968.4 [710.4–1266.0]	1003.9 [671.6–1404.0]	0.003
Refined foods	524.4 ± 475.2	706.7± 651.5	<0.001
**Carbohydrate (g)**			
Whole foods	56.6 [41.7–79.3]	61.1 [43.2–94.2]	0.611
Refined foods	85.6 ± 81.0	116.8 ± 111.5	<0.001
**Protein (g)**			
Whole foods	36.5 [27.4–47.0]	41.6 [29.2–62.6]	0.013
Refined foods	12.7 ± 11.6	18.1 ± 17.2	<0.001
**Fat (g)**			
Whole foods	61.8 [41.2–87.9]	61.5 [40.0–86.6]	<0.001
Refined foods	14.2 ± 14.2	17.9 ± 18.0	0.005
**Soluble Fiber (g)**			
Whole foods	5.2 [3.6–7.9]	5.1 [3.2–8.3]	0.143
Refined foods	1.8 ± 1.7	2.7 ± 2.7	<0.001
**Insoluble Fiber (g)**			
Whole foods	2.6 [1.8–3.7]	2.6 [1.5–3.9]	0.345
Refined foods	2.0 ± 2.0	3.0 ± 3.1	<0.001

Values are presented as median (IQR) or mean ± standard deviation (SD), as appropriate. Differences between groups were analyzed using the Mann–Whitney U test or independent samples *t*-test, as appropriate. A *p*-value < 0.05 was considered statistically significant.

**Table 5:** Correlations between dietary fiber intake and glycemic control parameters.

Variable (*n* = 820)	HOMA-IR	Glucose (mg/dL)	Insulin (µIU/mL)	HbA1c
	*r*/*p*	*r*/*p*	*r*/*p*	*r*/*p*
**Whole Food Fiber (g)**	−0.094/0.024	0.055/0.167	−0.099/0.015	0.034/0.581
Adj. *p*-value ^2^	0.096	0.445	0.096	0.868
**Total Fiber (g)**	−0.013/0.747	0.036/0.362	−0.011/0.796	0.010/0.868
Adj. *p*-value ^2^	0.868	0.724	0.868	0.868

^2^ Adjusted *p*-values were calculated using the Benjamini–Hochberg false discovery rate (FDR) procedure to account for multiple comparisons.

**Table 6:** Multiple Linear Regression Analysis of Total Fiber Intake and Log-Transformed HOMA-IR $(\log_{10}\mathrm{HOMA}-\mathrm{IR})$.

	B	SE	β	T	* p *	95% CI
(Constant)	−0.260	0.132	—	−1.969	0.049	[−0.519, −0.001]
Total Fiber (g/day)	−0.014	0.005	−0.148	−2.723	0.007	[−0.024, −0.004]
Age (years)	−0.001	0.002	−0.024	−0.534	0.593	[−0.005, 0.003]
Sex	0.053	0.048	0.044	1.099	0.272	[−0.042, 0.147]
Total Energy Intake (kcal)	0.000	0.000	0.055	1.005	0.315	[<0.001, <0.001]
BMI (${\mathrm{kg}/m}^{2}$)	0.041	0.005	0.378	8.442	<0.001	[0.032, 0.051]

$R^{2}=0.158$, Adjusted $R^{2}=0.151$, $F\left(5,537\right)=20.224$, p<0.001. Dependent Variable: Log-transformed HOMA-IR ($\log_{10}\mathrm{HOMA}-\mathrm{IR}$). Raw HOMA-IR values were log-transformed prior to analysis to satisfy normality assumptions. B: Unstandardized regression coefficient; SE: standard error; β: standardized regression coefficient; CI: confidence interval; BMI: Body Mass Index.

**Table 7:** Correlations of dietary fiber intake with visceral fat area and body mass index.

Variable	Visceral Fat Area (cm^2^) Female (*n* = 569)	Visceral Fat Area (cm^2^) Male (*n* = 251)	BMI (kg/m^2^)
	*r*/*p*	*r*/*p*	*r*/*p*
Whole Food Fiber	−0.050/0.290	−0.127/0.071	0.016/0.667
Total Fiber (g)	−0.008/0.865	−0.109/0.122	0.047/0.211

Values are Spearman correlation coefficients (r) with corresponding *p* values.

## Data Availability

The data presented in this study are available on reasonable request from the corresponding author. The data are not publicly available due to ethical and privacy restrictions related to participant confidentiality.

## Supplementary Materials

The following supporting information can be downloaded at https://www.mdpi.com/article/10.3390/nu18162698/s1. Figure S1: Flow diagram of participant recruitment, exclusions, and inclusion in the final analysis.

## Abbreviations

The following abbreviations are used in this manuscript:

BMI	Body mass index
EFSA	European Food Safety Authority
FDR	False discovery rate
FFQ	Food Frequency Questionnaire
HbA1c	Glycated hemoglobin
HOMA-IR	Homeostatic Model Assessment of Insulin Resistance
SPSS	Statistical Package for the Social Sciences
TBSA	Turkey Nutrition and Health Survey
TÜBER	Turkish Dietary Guidelines
eBeBİS	Nutrition Information System
NHANES	National Health and Nutrition Examination Survey
