# Supplementary material for: Dietary Fiber Intake, Food Sources, and Their Associations with Metabolic Parameters in Adults
Source: Nutrients. 2026 Aug 18;18(16):2698. doi: 10.3390/nu18162698 (PMC13516419; doi:10.3390/nu18162698)
Supplement: Supplementary file 1 [file nutrients-18-02698-s001.zip › nutrients-4448426-supplementary.pdf]

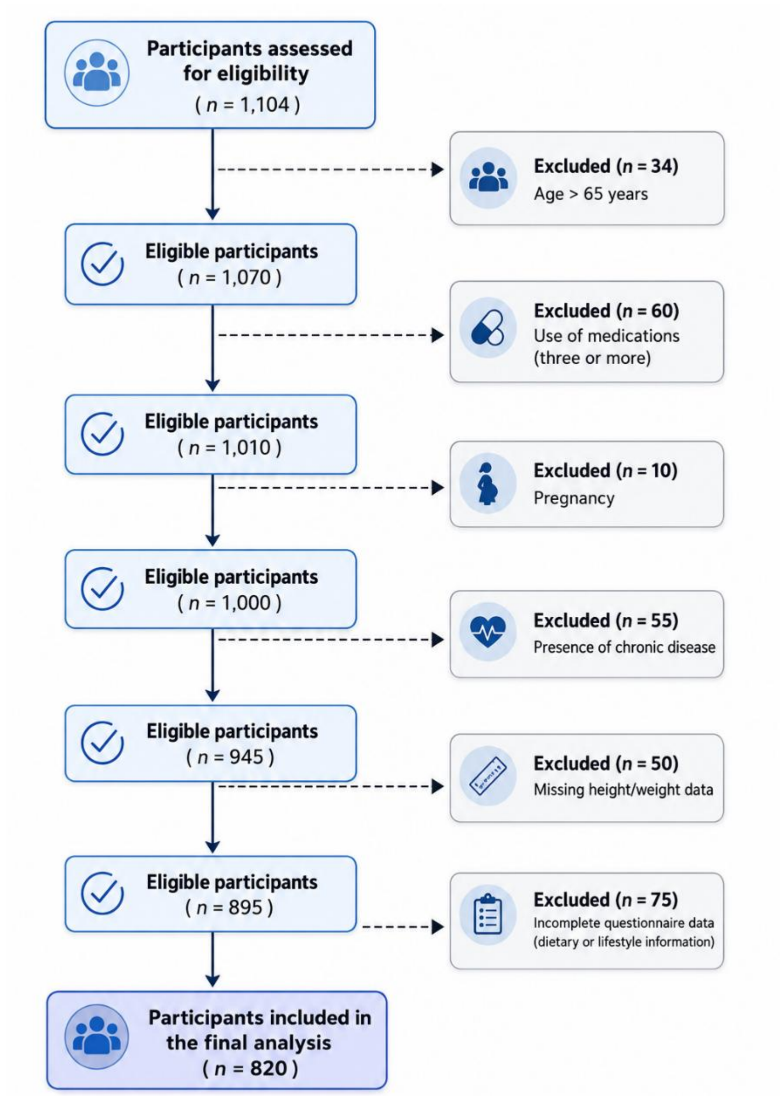

Figure S1. Flow diagram of participant recruitment, exclusions, and inclusion in the final analysis.
